# Empowering Rural Communities to Measure Walkability: Co‐Development of a Digital Tool

**DOI:** 10.1111/hex.70661

**Published:** 2026-04-09

**Authors:** Verity Cleland, Sharon Campbell, Georgia McGrath, Claire Morley, Melanie Davern, Anna Timperio, Kate Garvey, Yvonne Laird, Samantha Rowbotham, Lynden Leppard, Kim Jose

**Affiliations:** ^1^ University of Tasmania Hobart Tasmania Australia; ^2^ RMIT University Melbourne Victoria Australia; ^3^ Deakin University Melbourne Victoria Australia; ^4^ Tasmanian Department of Health Hobart Tasmania Australia; ^5^ University of Sydney Sydney New South Wales Australia; ^6^ Local Government Association of Tasmania Hobart Tasmania Australia

**Keywords:** built environment, citizen science, engagement, neighbourhoods, physical activity, regional

## Abstract

**Background:**

Local neighbourhood environments are important for shaping walkability, but few instruments exist to assess walkability in rural areas. Furthermore, there are no existing tools that have been designed with and for communities to collect local data on walkability, which has the potential to empower communities with ownership and knowledge of the resulting information.

**Objective:**

This paper aimed to describe the development and evaluation of a digital tool designed to measure walkability in rural areas.

**Methods:**

The Communities for Walkability (C4W) digital tool was co‐developed by researchers and community members. Sixty‐one community members in 10 small rural Tasmanian towns conducted 80 street segment audits and/or participated in workshops; 16 participants engaged in semi‐structured interviews. Geospatial assessments of walkability were completed using Geographic Information Systems (GIS). Qualitative data were content/thematically analysed and quantitative data analysed descriptively. Exploratory analysis was performed to assess correlations between geospatially assessed walkability and a range of audit scores.

**Results:**

It was feasible for citizen scientists to collect data using the largely acceptable digital tool, with some areas for improvement identified, particularly the conflict between the sequential nature of tool items and the non‐sequential nature of attributes encountered during data collection, the inability to review/save findings, and technical difficulties uploading photographs. Exploratory analysis revealed some correlations between geospatially assessed walkability and safety, commercial land use, and perceived overall walkability; however, the small sample size and exploratory nature of analyses suggest cautious interpretation.

**Conclusions:**

Although feasible and acceptable for rural communities to collect walkability data using a citizen science approach, future digital walkability tools should allow greater flexibility for real‐time data collection, provide streamlined systems for uploading photographs, and include review and save features. The inclusion of citizen perspectives in the design and collection of local data has the potential to be a powerful mechanism to support communities and stakeholders to engage in collective efforts and create environments that support walkability.

**Patient or Public Contribution:**

Members of the public were involved in this study in four key ways: co‐development of the digital walkability tool, data collection using the digital walkability tool, sense‐making and interpretation of findings from the digital walkability tool, and through interviews providing feedback on the tool.

## 1 Introduction

While a substantial evidence base has established the importance of neighbourhood design for enabling physical activity [1, 2, 3, 4, 5], this research has focused on large urban city environments, with little attention to rural and regional areas. Those living in rural and regional areas tend to have poorer health outcomes and are less active than urban dwellers. For instance, compared to those living in urban Australia, hospitalisations and death related to cardiovascular disease are around 40% higher [6] and Type 2 diabetes up to 30% higher [7] among those living rurally, and rural residents are less active [1]. Similarly, in the United States (US), age‐adjusted death rates for cardiovascular disease, stroke, diabetes, and cancer—all conditions associated with low physical activity—are higher in rural than urban areas [2]. In Australia, only 24% of Australians aged over 15 years meet physical activity guidelines [7], and similar urban‐rural inequities are observed in many other developed countries [3, 4, 5]. Given that physical activity is protective against these most common and costly diseases, it is important to identify factors that positively influence physical activity behaviour in rural populations.

Our qualitative work has found important nuances around rural environment attributes, with rural residents noting similar overarching environmental constructs to urban areas (e.g., ‘safety’) but differences in conceptualisation (e.g., trip hazards and wildlife in rural areas vs. safety from crime in urban areas) [8, 9]. A 2010 systematic review of studies assessing the built environment and physical activity in rural areas concluded there was limited research (mostly from North America) and recommended development and refinement of new tools to measure local environments in rural contexts [10]. Progress in understanding local environment‐physical activity relationships has been slow and limited by the lack of environment measurement instruments developed specifically for rural populations.

Citizen science (i.e., engagement of community members in gathering and making sense of data) is an emerging area of research that offers a unique approach to simultaneously engage communities in research, empower community voice, influence local planning decision‐making, and collect locally relevant data. Citizen science may be particularly suited to research in rural communities where data collection may be difficult due to geographic dispersion, expense or time constraints [11]. Having community members collect data on their local neighbourhoods also provides greater lived experience insights and may facilitate engagement in advocacy activities to improve and support physical activity and wellbeing. While a number of community‐oriented tools are available for community members to audit aspects of their local environments (e.g., Heart Foundation of Australia's Community Walkability Checklist; the US National Department of Transportation's Walkability Checklist [12, 13]), few have been developed for rural areas.

Digital tools are increasingly being used in citizen science projects to activate and support communities to engage in scientific processes, with a recent systematic review identifying 62 articles for citizen science applications that support digital (web or mobile) data collection [14]. This paper describes the development and evaluation of the Communities for Walkability digital tool, a resource for communities to measure walkability in rural areas. While there are many definitions of walkability, we consider walkability to be the ‘natural, built, and social properties of neighbourhoods that promote physical activity and health and allow for equitable access to health‐enhancing resources’ [15]. The specific objectives of this mixed methods study were: (1) describe the development of the C4W digital tool; (2) describe the community experience of using the C4W digital tool; and (3) an exploratory analysis to compare data collected from the C4W digital tool with geospatially assessed measures of walkability.

## 2 Materials and Methods

The Communities for Walkability (C4W) study (https://walkrural.com.au/) was approved by the University of Tasmania Human Research Ethics Committee (3 March 2021; approval 23174). All participants were aware of the goals of the study and provided informed consent. We followed the COREQ guidelines for qualitative research [16] (see Supporting Information S1: Table S1) and the STROBE guidelines for cross‐sectional research [17] (see Supporting Information S1: Table S2). Interviewers and facilitators approached interviews from a place of curiosity and openness to new ideas and were guided by open‐ended questions to support discussion.

### 2.1 Study Design and Context

The C4W mixed methods study was conducted across Tasmania, Australia, which is 68,401 km^2^ (42,502 m^2^) and in 2021 had 557,571 residents (population density: 8.15/km^2^) [18]. It is the only Australian state with no urban area classified as a ‘Major city’ and hence is entirely regional/rural [19]. The population is geographically dispersed, and typically clusters across south, north, and north‐west regions. Most reside in three major centres: Hobart (south; 247,100), Launceston (north; 91,000), and cluster of Burnie, Wynyard, Devonport and Ulverstone (north‐west; 74,600), supplemented by smaller but economically important regional centres [18]. The remaining 157,000 residents are scattered across small towns and settlements.

The C4W study builds on a 2020 pilot study that, in response to needs expressed by local and state government authorities, assessed the feasibility of using a citizen science approach to collect walkability data in small rural towns [20]. In three pilot study towns, we developed a ‘pen and paper’ audit tool using items from the Rural Active Living Assessment (RALA) tool [21], a suite of three instruments to measure active living environments in rural communities, including street segments, a townwide assessment, and a town policy/programme assessment. RALA is evidence‐informed, with demonstrated feasibility and reliability [21]. It has been used in across the United States, including in Alabama and Mississippi [22], Texas [23], Latino communities in Central Washington [24], and in northwestern North Carolina [25], but only by researchers [23, 24, 25] or highly trained community workers [22]. Our pilot study indicated that it was feasible for community members to collect walkability data, but some items required further contextual adaptations and participants indicated a desire for a digital version for ease of use [20].

Phase 1 of the C4W study involved geospatial mapping of 92 rural Tasmanian towns (population size: 200–6000) to generate three individual indices and one summary index of walkability [26, 27]. Phase 2 involved (a) development of a digital tool, (b) working closely with 10 purposefully selected communities to conduct audits, (c) discussing findings in community workshops, and (d) semi‐structured interviews about participation. Phase 3, not reported here, involved developing town reports that incorporated data from Phases 1 and 2 to highlight key issues and priorities for action. We also provided advocacy capacity building support to all participants (e.g., resources, templates, training).

### 2.2 Phase 1: Geospatial Mapping

Using the Australian Bureau of Statistics (ABS) Stories from the Census, 2016 Small Town list for Tasmania [28], we identified 92 towns for geospatial mapping. At the Mesh Block level (the smallest geographical unit of the Australian Statistical Geography Standard used by the ABS [29]), neighbourhood attribute scores were calculated for street connectivity (ratio of intersections to local walkable neighbourhoods, square kilometres), dwelling density (total dwellings within 1600 m of a road network), and daily living destinations (presence/absence of supermarkets, public transport stops, convenience stores with 1600 m of sample points). A summary walkability index was calculated as the sum of standardised scores of the three attributes, weighted by Mesh Block population [30].

### 2.3 Phase 2: Data collection

#### 2.3.1 Town Selection

**Figure 1:**
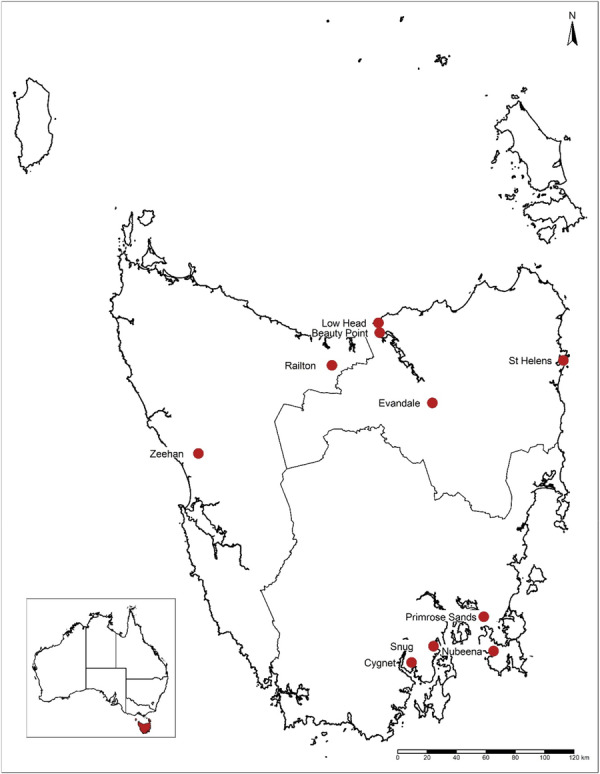
Towns recruited for the Communities for Walkability project in the south, north, and north‐west regions of Tasmania, Australia.

To select towns, the summary walkability index of the 92 small Tasmanian towns identified in Phase 1 were ranked into deciles. We excluded towns with a population size < 400 residents (*n* = 29) as our pilot study indicated feasibility issues with smaller towns [20]. We also excluded towns that participated in our pilot study (*n* = 2), towns that to our knowledge were currently or recently involved in other related research projects (*n* = 2), and towns deemed an ‘outer suburb’ of a larger centre (*n* = 1). We then worked with local and state government policy partners to consider community interest, engagement or need, and to reflect diversity across Tasmania, local government areas, and town size. This led to the identification of a primary and replacement town from each decile, with the goal of recruiting one town per walkability decile to participate in Phase 2 (10 towns in total; Figure 1).

### 2.4 Recruitment

Recruitment involved working with policy partners and local networks to identify one or more ‘Community Champions’ in each town; this was seen as an important first step in building trust and tapping into existing networks. Because the broader C4W project had a focus on community empowerment, community capacity building and advocacy, this approach reflects community development principles including providing leadership roles to people in the community, working with rather than for the community, helping to recognise skills, knowledge, and expertise, encouraging working collectively rather than individually, learning by doing, and promoting social justice and respect [31]. Community Champions identified community members (‘Citizen Scientists’) to participate. Staged recruitment began in October 2021 and finished in June 2023.

#### 2.4.1 Community Champions

Eligibility criteria were: aged 18+ years; a member of the community; have knowledge of and involvement in the community; have or are willing to apply for a ‘Working With Vulnerable People’ registration with the Tasmanian government; and willing and able to fill in checklists about the community, help recruit citizen scientists, and support community workshops. Community Champions undertook a 1‐h online training session with an investigator (VC, *n* = 9 sessions; KJ, *n* = 1 session) and the Project Manager. The content included project background, citizen science, ethics, roles and expectations, safety, taking photographs, the digital tool, and community workshops, and questions and discussion.

Once identified, the Project Manager granted the Community Champion access to the C4W digital tool via an email login link. A participant information sheet and online consent were required to proceed. Community Champions then accessed the dashboard and completed the About You, Town Wide Assessment, and Program and Policy Assessment sections. Community Champions used the digital tool (independently or with support from the Project Manager) to self‐identify town segments along the road network for auditing, and to recruit Citizen Scientists to complete segment audits. Community Champions were instructed to choose segments that were approximately 1–1.5 km in length and that they considered meaningful and relevant to the study's aims. There were no other criteria for selecting segments or for the minimum or maximum number of segments in each town.

#### 2.4.2 Citizen Scientists

Eligibility criteria were: aged 12+ years; living or working in the town; and willing and able to fill in checklists about the community. Parental/guardian consent was required for Citizen Scientists < 16 years old. An email login link was sent by the Project Manager or Community Champion, with a participant information sheet and online consent required to proceed. Citizen Scientists could then access the dashboard and complete About You and Segment Assessment sections. Citizen Scientists < 16 years were encouraged to complete the audit with an adult.

### 2.5 Community Experience of the C4W Digital Tool

To address our second objective, we extracted data from: (1) community workshops that occurred as part of sense‐making and priority setting, and (2) semi‐structured interviews with Community Champions and Citizen Scientists once data collection and reporting were complete.

#### 2.5.1 Community Workshops

Community Champions, Citizen Scientists, and other interested community members and stakeholders were invited to attend a workshop in each town as soon as practicable after audits were completed (usually within 3 months). Each 1.5–2‐h workshop was held at a local community venue (e.g., community hall) and was facilitated by at least one member of our research team (KJ: PhD‐trained woman with 20+ years' experience in facilitation, project investigator; GM: PhD‐trained woman with 4 years' experience in facilitation, project manager), supported by a research assistant. The discussion was informed by the audit data and photographs to identify key issues and priorities within each community. Specific questions seeking feedback about the C4W digital audit tool were asked.

#### 2.5.2 Semi‐Structured Interviews

All Citizen Scientists and Community Champions were invited to participate in a semi‐structured interview, conducted by one of two members of our research team (CM PhD‐trained woman with 10 years' experience in interviewing, project manager; GM: PhD‐trained woman with 7 years' experience in interviewing, research fellow). Overall, 12 Community Champions and four Citizen Scientists from nine towns took part between December 2022 and April 2024; interviews lasted on average 32 min (range: 18–54 min). Interviews were conducted for the primary purpose of understanding the general experience of involvement across the whole project (e.g., reasons for participating, expectations, challenges, etc.) but participants were asked about: the experience and challenges in using the tool; what worked well and what did not; what could be improved to make the tool easier to use; perceptions of how the tool would be used by Citizen Scientists; and whether the tool would be easy for all community members to use (e.g., children, older people, people with limited mobility).

### 2.6 Data Analysis

Recordings of community workshops and interviews were transcribed, compiled and analysed in qualitative data management software QSR NVIVO (Version 16.1). Transcripts underwent a process of coding, categorisation and thematic analysis by experienced qualitative researchers CM and GM with input from senior qualitative researcher KJ. The analytic team (VC, KJ, CM, GM) met regularly to review the coding and discuss key themes. Following this, a codebook of descriptions was created.

A narrative is provided to describe the development of the digital tool (Objective 1). To describe digital tool data (Objective 2), means and ranges (continuous data) and percentages (categorical data) were calculated. To address our third objective, Spearman correlations were calculated for audit tool data from segment assessments and were plotted against geospatially assessed walkability decile. Spearman correlation coefficients were calculated between both the town‐wide assessment tool and geospatially assessed walkability decile and population size, and Spearman correlation coefficients were also calculated between the programme and policy tool and geospatially assessed walkability decile. Note that the correlation coefficients presented are a general indication of the direction and magnitude of relationships but should be interpreted with caution due to the very small sample size. All quantitative analyses were conducted in R v4.2.2 (The Comprehensive R Archive Network [Software] CRAN Network 2023 https://cran.r‐project.org/).

In this mixed methods study, data integration occurred through both connecting and embedding data to address the different study aims [32]. Data connection occurred to address Objective 1 (development of the digital tool) where modification of an existing audit tool followed by feedback from a Consumer Reference Group informed digital tool development and for Objective 2 (community experience of using the digital tool) where geospatial walkability assessments enabled towns to be categorised into deciles to inform town selection. Further data integration occurred for Objective 2 with data collection about the experience of using the digital tool being embedded in workshop discussions. Objective 3 involved a direct comparison between two quantitative data collection approaches.

## 3 Results

Findings are presented for each objective as follows: (1) description of digital tool development; (2) town selection, characteristics of towns and study participants; (2) community experience of the C4W digital tool; and (3) exploratory comparison of C4W digital tool and geospatially assessed walkability data.

### 3.1 Digital Tool Development

The C4W digital tool was developed by a technology company and co‐designed through a collaboration between the research team, a Consumer Reference Group, and our policy stakeholders. The technology company was briefed on the project aim, tool requirements (e.g., web‐based interface, confidentiality, user rights, central database) and the project budget. With the increase in the number of citizen science projects internationally, inclusion of users in the design of digital tools to ensure engagement and usability is considered increasingly important for the sustainability of citizen science [33]. A Consumer Reference Group, established to co‐design the tool and provide general input into the C4W project, included members (*n* = 8) recruited from our pilot study [20] and through project team networks. The Consumer Reference Group comprised two young people (< 16 years) and six adults (age 18+ years) with diversity in age, gender, geographic region, and occupation, and with all but one living in one of the 92 small rural towns. The Consumer Reference Group met online for approximately 1 h on three occasions over a 1‐year period. These meetings mostly focused on the design and functionality of the digital tool. Consumer Reference Group members also provided ad hoc input into the development of the digital tool via email and telephone upon request. Once a prototype was developed, Consumer Reference Group members tested the tool in the field and provided feedback, which were incorporated by the technology company. Two policy stakeholders were also invited to review the tool.

**Figure 2:**
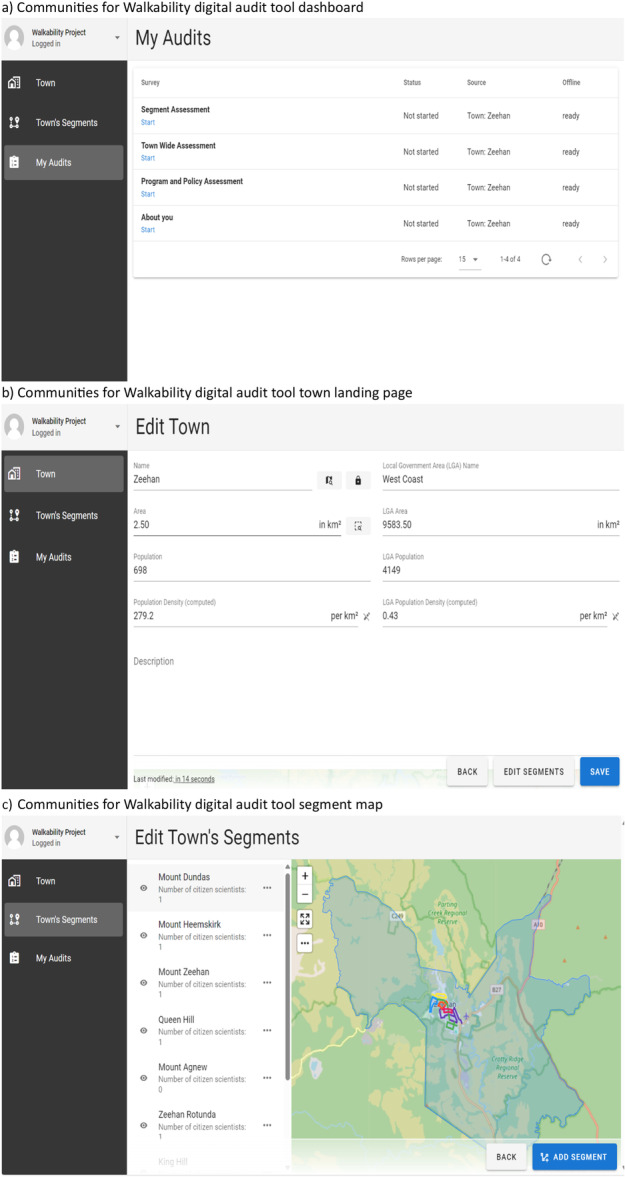
Communities for Walkability digital audit tool (a) dashboard, (b) town landing page, and (c) segment map.

The CW4 digital tool was adapted from the RALA instruments, with modifications informed by our pilot study and Consumer Reference Group, largely for clarity and local context (see Supporting Information S1: Tables S3–5 for full list of items, changes made, rationale for changes). The C4W digital tool was accessed via a web interface and was password‐protected (Figure 2a–c). It consisted of a dashboard with four separate sections: About You (10 items), Town Wide Assessment (25 items), Program and Policy Assessment (seven items), and Segment Assessment (24 items). Treatment and scoring of variables are summarised in Table 1. Consistent with RALA, the townwide and policy and programme sections were designed for completion by one person in each town. Segment Assessments were designed for completion by one or more users per segment. Each question gave users the option to provide other comments and upload photographs.

**Table 1:** Scoring of items in the C4W digital tool.

Assessment	No. of questions	Questions included in scoring	Scoring method
About You	10	n/a	n/a
Town Wide Assessment	25	n/a	n/a
Program and Policy Assessment	7	6	Sum of the following (where the presence of these items indicates a positive score): 1. council policy that requires bikeways or pedestrian walkways in new public infrastructure projects 2. regular clearance of obstacles from footpaths 3. council community recreation department that offers physical activity programming 4. physical activity resources or facilities available for local resident use outside of programs 5. “Walk to School” programs or other programs that encourage children to walk or bike to school 6. schools that allow public access to their recreation facilities after school hours
Street Segment Assessment	24*	14	
General walkability and accessibility	4	4	Sum of: 1. general walkability 2. accessibility (e.g. pram or elderly) 3. walkability for children 4. general aesthetics
Public and commercial land use	2	2	Sum of: 1. mean no. of commercial land use features per segment (e.g. restaurant/café, pub/bar/nightclub, supermarket, small food store, farmers market, theatre/cinema, petrol station, general store, small retail shop, large retail shop, gym/fitness centre, private medical office, private other office, other) 2. mean no. of public land use features per segment (e.g. library, online access centre, museum, community centre, community hall, men's shed, post office, town offices, court of law, police station, fire station, church/religious centre, hospital/health care, sports field, indoor/outdoor sports centre, playground, public toilet, public park, other public open space, other)
Safety characteristics	3	3	Sum of: 1. presence and condition of footpath buffers and defined curb shoulders 2. presence and condition of crossings and signage, including zebra (pedestrian) crossings, crossing signals, pedestrian signs, children at play signs 3. other safety features (presence of traffic lights, stop signs, school flashing lights, slow down signs, school crossings guides, speed bumps, chicanes, public lighting, ramps on footpath gutters)
Walkability barriers	1	1	Mean no. of barriers per segment, all reversed scored (e.g. highway crosses over/impacts, train tracks cross over/impacts, private property/no trespassing impacts, industrial zones, natural features, roundabouts, others)
Walkability enablers	4	4	Sum of: 1. presence and condition of footpaths (scored higher when present on both sides of the street and in good condition, lower when not present/poor condition) 2. traffic volume (scored higher when low volume) 3. tree shade (scored higher when trees present on both sides, lower when no trees) 4. presence and condition of connectors (scored higher when segments linked to other attractions or segments)

*Note:* *10 Questions were not included in the scoring matrix, including (but not limited to) those on terrain, segment zone type, weather conditions, season and day of week (see Supporting Information S1: Table S3).

### 3.2 Characteristics of Towns and Study Participants

Town recruitment was successful in all but one primary town, where a Community Champion could not be identified, and hence the reserve was used. Two towns had initially been excluded from our shortlist due to their population size. However, we were approached by two residents from these adjoining towns who argued that the towns should be considered together, as they were in close proximity and local residents typically consider these as one area. Due to the purposive nature of our sampling approach and the clear engagement, interest, and potential community benefit, they were included. However, it should be noted that these towns were from different walkability deciles, so are excluded from some of the quantitative analyses.

The 10 towns ranged in size from 465 to 1560 residents, with a mean population of 806 (Table 2). The average number of segments audited per town was 7.3, with a total average of 8831 m per town (range: 4777–13,497 m) audited. Average segment length was 1224 m (range: 796–1844 m).

**Table 2:** Town and segment audit characteristics.

Town	Resident population	No. of segments	Segment distance min (m)	Segment distance max (m)	Segment length mean (m)	Segment distance total (m)
1	835	9	877	2178	1500	13,497
2	1171	6	841	1311	1099	6592
3	929	8	792	1398	1174	9390
4	561	10	563	1509	1059	10,062
5	472	8	577	1143	882	7059
6	1050	9	741	1485	1013	9114
7	1124	9	835	1195	1076	9682
8	465	7	756	3308	1368	9573
9	698	6	1456	2769	1844	11,061
10	1560	8	877	2411	1389	11,112
Total	8865	80	—	—	—	97,142
Average	806	7.3	863	1817	1224	8831

Table 3 describes the characteristics of C4W participants who used the digital audit tool. Participants more commonly were female (62%), aged 35+ years, non‐students (83%), university educated (53%), employed (43%) or retired (42%), had no children in the household (70%), had good/very good self‐assessed health (75%), did not require mobility aids (96%), lived in the town (96%) on average of 8 years, and those who worked in the town (42%) had done so for nearly 7 years.

**Table 3:** Characteristics (%) of participants and community champions who used the digital audit tool in the C4W study.

Characteristic	All participants (*N* = 53)	Community champions (*N* = 13)
Gender, *n* (%)		
Male	18 (34.0)	1 (7.7)
Female	34 (62.2)	12 (92.3)
Non‐binary/other	1 (1.9)	0
Unknown	0	0
Age, *n* (%)		
< 18	5 (9.4)	n/a
18–24	0	0
25–34	0	2 (15.4)
35–44	12 (22.6)	4 (30.8)
45–54	8 (15.1)	2 (15.4)
55–64	12 (22.6)	3 (23.1)
65+	16 (30.2)	2 (15.4)
Student, *n* (%)		
Yes full‐time	5 (9.4)	0
Yes part‐time	4 (7.5)	4 (30.8)
No	44 (83.0)	9 (69.2)
Education, *n* (%)		
Year 10 or less	6 (11.3)	0
Years 11–12 or equivalent	2 (3.8)	0
Trade certificate, apprenticeship or diploma	17 (32.1)	3 (23.1)
University degree	22 (41.5)	6 (42.6)
Postgraduate degree	6 (11.3)	4 (30.8)
NA	0	0
Labour force status, *n* (%)		
Employed	23 (43.4)	8 (61.5)
Not in the labour force	3 (5.7)	0
Student	4 (7.5)	0
Retired	22 (41.5)	5 (38.5)
Unknown	1 (1.9)	
Children in household, *n* (%)		
Yes	16 (30.2)	6 (46.2)
No	37 (69.8)	7 (53.8)
NA		
Self‐assessed general health, *n* (%)		
Excellent	9 (17.0)	5 (38.5)
Very good	20 (37.7)	2 (15.4)
Good	20 (37.7)	3 (23.1)
Fair	4 (7.5)	3 (23.1)
Poor	0	0
NA		
Mobility aids, *n* (%)		
Yes	2 (3.8)	0
No	51 (96.2)	13 (100)
Lives in town, *n* (%)		
Yes	51 (96.2)	11 (84.6)
No	2 (3.8)	2 (15.4)
Length of time lived in town in years, mean (range; SD)	8.25 (0.8–38.0; 7.6)	7.3 (0.25–31.0; 9.13)
Work in town, *n* (%)		
Yes	22 (41.5)	5 (38.5)
No	31 (58.5)	11 (84.6)
Length of time worked in town in years, mean (range; SD)	6.85 (0.1–20; 6.2)	6.0 (1–10; 3.7)

The digital tool was able to effectively collect segment audit data for towns (Supporting Information S1: Tables S6–7). Individual items from segment assessments are described in Supporting Information S1: Table S8.

### 3.3 Community Experience of the C4W Digital Tool

When assessing the acceptability of the digital tool, several participants indicated it was easy to use (e.g., *“Couldn't imagine having done it [the survey] without it*…*nice, neat, simple, easy”*). Some indicated they had to invest time to familiarise themselves with the tool *“I needed the time to sit down and make friends with the online tool”*, but once they did, they were able to use the tool effectively:

It took me a couple of days to work it out, but eventually I found out how to do it.

Not everyone agreed the tool was easy to use, with some participants indicating it was *“clunky”* and one had particularly strong feelings *“I hate your technology”*. There were some minor concerns about accessibility (*“It doesn't come up on the phone very well, just the screen size and stuff like that”*) and seemingly random ‘glitches’. The three main issues for participants were photo uploads, the sequencing of items, and the lack of options to review or save data.

Photographs could be uploaded alongside any question in the tool, but this was not apparent to some:

“… I took a heap of photos, but I didn't use all of them because some of the photos didn't – I was trying to get them to relate to the question that they were joined to and I couldn't always do that, which I found a bit frustrating. So a lot of my photos weren't included because I couldn't get them to fit in with the – put them in context.”

The sequential nature of the C4W digital tool was frustrating for some participants as they were unable to anticipate the type of questions they were going to be asked; questions focused on the whole segment rather than different sections. Many found workarounds to this by taking notes or printing paper copies then entering data later. These frustrations and workarounds are captured in the reflections of this Citizen Scientist:

I set off to do it on my phone, but the questions actually linear like that, they don't apply to the first part of your walk and then the second part of your walk, the third part of your walk, I was sort of being asked questions about the whole walk, you know, the context of the area and things like that, that I couldn't answer in a kind of linear walking kind of way… I couldn't see what was coming, so I didn't know, do I go into depth here or are they going to ask me another question here or not? And I didn't know what was happening. When I realised what was going on, I just took lots of photos on my phone and then I got back to my computer and uploaded them onto the computer so I could then sit down and do the survey on my computer…

Another concern was that participants were not able to review data prior to submitting, and could not save a copy of the data for future reference, which for some undermined their contribution:

And then the last sort of terrible thing was, when I finished the last question, I could never see it again. I couldn't even see the finished thing and I certainly couldn't edit it… Gone. It's evaporated…

### 3.4 Comparison of Data From the C4W Digital Tool and Geospatially Assessed Walkability Index

Results for exploratory correlation analysis between geospatially assessed walkability and various audit variables are presented in (Supporting Information S1: Table S9 and Figures S1–6). Commercial land use features and ‘other’ safety features were positively significantly correlated with geospatially‐assessed walkability (0.61 and 0.71), and a number of other audit variables (overall walkability, accessibility for older adults, buffers and should, all safety features, footpaths) were correlated at moderate levels and in the expected direction (Spearman's *r* = 0.40–0.48), but were not statistically significant. Given the small sample size for this analysis, these exploratory findings should be interpreted with caution.

## 4 Discussion

This mixed methods study aimed to describe the development and evaluation of a digital assessment tool designed with and for communities to measure walkability in rural areas. It was feasible to develop a digital tool in a partnership between researchers, policy stakeholders, community members, and a technology company, with 66 community members in 10 towns auditing 80 street segments totalling 97 km. While the tool was generally considered acceptable, some improvements were identified to enhance the experience. Exploratory correlational analysis between the audit tool and geospatially assessed walkability showed correlations in the expected directions, with safety and commercial land use features showing the strongest correlations. Combined, these findings suggest that the C4W digital tool was generally a feasible way to collect information on walkability in rural areas, but modifications to improve the user experience are warranted prior to further use.

A common criticism of the tool was its setup as a linear online survey. While navigational buttons allowed participants to move back and forward through individual pages (one question per page), the sequential design conflicted with the non‐sequential physical process of auditing a street segment, where there is substantial variability in the features that would be encountered while moving through a segment. This conflict made it difficult for users to perform the audit in real time, and while some found this frustrating, many did not and most who did were able to identify acceptable workarounds. Some took notes and photographs during the audit for upload later, while others printed paper copies for their fieldwork. This latter option was offered by the project team at the outset of the project, as we were aware that digital literacy and internet connectivity may pose participation barriers, but few took that opportunity. Some participants expressed disappointment at being unable to review and/or save their responses at the completion of the audit, and as a result, we added this feature to the digital tool (meaning participants in the later phases of the study were able to review and save results). In line with citizen science principles [34], future iterations of the digital tool could consider making individual and collectively gathered data publicly available. Providing greater and clearer anticipatory guidance, providing an overview at the beginning of each section, and providing navigational menus or a dashboard‐style home page could be considered in future iterations.

Photo elicitation methodologies have been commonly used in anthropology and sociology, and increasingly in health research, as an additional communication tool and to generate discussion [35]. In this project, photographs were collected by participants to demonstrate barriers and enablers to walkability, and to prompt discussion in community workshops. Participants could upload photographs alongside each question in the digital tool to illustrate responses. Several participants had issues uploading photographs. Some difficulties were resolved by the research team, but others were related to the tool's design and were unresolvable. Also noted was confusion about uploading photographs alongside individual questions, indicating clearer guidance is required in future iterations and more comprehensive user experience testing during development. Despite its co‐development with a Consumer Reference Group and partners and pilot testing, not all issues were identified, likely a result of the extensive variation in user capabilities and vast differences in devices (e.g., phone, computer, tablet) and their age, brand, and operating system. However, we did not collect information on the type, brand, model, operating system, or age of digital devices used by participants so are unable to confidently ascertain this as the cause of these issues. The development of a digital walkability assessment tool within a research project context, with associated budget constraints, precluded more extensive user experience testing.

While the C4W digital tool and the geospatial assessments of walkability measure different aspects of walkability, we anticipated some correlation between the two measures. For instance, two North American studies using the RALA tool found associations between the subjective reports of overall walkability with sidewalks, street shoulders, and connectivity [24, 25], public/civic buildings and safety features [25], and physical activity amenities [24]. Despite warranting caution in interpretation due to the small sample size, data from our exploratory comparative analysis suggested some consistency in results and insights that should be considered in future walkability research. The strongest correlation observed was between geospatially assessed walkability and the audit tool measure of ‘other safety features’ (*r* = 0.71), and the third strongest correlation was with ‘total safety features’ (*r* = 0.48). This finding is similar to a North American study that used subjective measures of walkability [25]. ‘Other safety features’ included the presence of traffic lights, stop signs, school flashing lights, slow down signs, school crossings guides, speed bumps, chicanes, public lighting, or kerb ramps on footpath gutters. ‘All safety features’ included these ‘other safety features’ items (likely driving the correlation) as well as the presence and condition of road buffers and shoulders, zebra crossings, crossing signals, pedestrian signs, and ‘children at play’ signs. These safety features may be more common in areas where people come together in towns by car, foot or bike, such as around shops and school, aligning with previous work noting the importance of safety in the rural context [8, 9], and to the compact urban conceptualisation of walkability. The presence of more daily living destinations captured by the geospatial assessment is also likely to explain the moderate correlation (*r* = 0.61) with commercial land use features captured in the digital tool.

### 4.1 Strengths and Limitations

The project was limited to 10 purposively selected rural Tasmanian towns, and while we aimed to maximise variation by selecting one town from each of 10 geospatially assessed walkability index deciles, findings may not be generalisable to rural towns in other jurisdictions or countries. Assessments were subjective and therefore potentially influenced by the characteristics of those who completed them. While not intended to be representative, compared to other residents from the study towns who had completed the 2022 Tasmanian Population Health Survey, participants conducting segment audits over‐represented females and those aged 35–44 years, those university‐educated, employed, and with excellent/very good health, and under‐represented those aged 65+ years, with lower levels of education, not in the labour force or retired, and with poorer health. Further, those volunteering to participate in this study may have been more interested in walkability, physical activity, and/or health, and hence more attuned to or aware of local conditions than others. Reliability testing is warranted. Not all streets in all towns were audited; other barriers or enablers to walkability may have been identified in other parts of towns not assessed. User difficulties tool may have impacted the findings, although most participants adopted acceptable workarounds and issues tended to be technological rather than assessment related. Correlations between a geospatially assessed walkability index and the C4W digital tool should be interpreted with caution due to the small sample size but provide a useful comparison.

A strength of this study was its mixed methods approach. We used geospatial assessments, a largely quantitative audit tool, photographs, group discussions, and interviews to evaluate the C4W digital tool. This breadth of approaches provides a richness of data that would be unattainable through one method alone and provides unique insights into the walkability of small rural towns and the strengths and limitations of the tool. The combined use of a systematic and purposive approach to sampling of towns ensured diversity in walkability (based on geospatial assessments) and in town characteristics (size, region, local government area, industry) but was also pragmatic in responding to community and policymaker needs. As was demonstrated in one town where we were unable to recruit a community champion, local community support is critical to successful implementation. A further strength was the collaboration with our Consumer Reference Group and policy stakeholders, with this type of involvement considered fundamental for effective data collection in the use and design of citizen science applications. Local government support in the project was a useful but not critical element in the identification and recruitment of participants.

## 5 Conclusion

This mixed methods study aimed to describe the development and evaluation of a digital tool designed with and for rural communities to measure walkability. The C4W digital tool demonstrated feasibility and was largely acceptable to users, with some improvements identified that need to be addressed and with further testing required before broader integration and upscaling into existing systems (e.g., local or state government). Digital tool measures of safety and commercial land use demonstrated correlations with geospatially assessed walkability in the expected direction, despite the small sample size. Future iterations of this and other digital tools for auditing walkability should allow greater flexibility in navigation between items to facilitate real‐time data collection in diverse environments, provide streamlined mechanisms for uploading photographs, and allow participants to review and retain data. The findings of this project suggest it is feasible and acceptable to use a digital tool for communities to collect data about walkability in rural towns.

## Author Contributions

**Verity Cleland:** conceptualisation, formal analysis, funding acquisition, methodology, project administration, supervision, writing – original draft preparation, writing – review and editing. **Sharon Campbell:** data curation, formal analysis, writing – review and editing. **Georgia McGrath:** data curation, formal analysis, methodology, project administration, writing – review and editing. **Claire Morley:** formal analysis, writing – review and editing. **Melanie Davern:** conceptualisation, funding acquisition, methodology, writing – review and editing. **Anna Timperio:** conceptualisation, funding acquisition, methodology, writing – review and editing. **Kate Garvey:** conceptualisation, funding acquisition, methodology, writing – review and editing. **Yvonne Laird:** conceptualisation, funding acquisition, methodology, writing – review and editing. **Samantha Rowbotham:** conceptualisation, funding acquisition, methodology, writing – review and editing. **Lynden Leppard:** conceptualisation, funding acquisition, methodology, writing – review and editing. **Kim Jose:** conceptualisation, formal analysis, funding acquisition, methodology, project administration, supervision, writing – review and editing.

## Ethics Statement

The Communities for Walkability study was approved by the University of Tasmania Human Research Ethics Committee (3 March 2021).

## Conflicts of Interest

The authors declare no conflicts of interest.

## Supporting information


Supporting File

## Data Availability

The data that support the findings of this study are available from the corresponding author upon reasonable request.
