# Supplementary material for: Empowering Rural Communities to Measure Walkability: Co‐Development of a Digital Tool
Source: Health Expect. 2026 Apr 9;29(2):e70661. doi: 10.1111/hex.70661 (PMC13066715; doi:10.1111/hex.70661)

**Supplementary Table 1- Consolidated criteria for reporting qualitative studies (COREQ): 32-item checklist**

| **No. Item** | **Guide questions/description** | **Reported on Page #** |
| --- | --- | --- |
| **Domain 1: Research team and reﬂexivity** |  |  |
| *Personal Characteristics* |  |  |
| 1. Interviewer/facilitator | Which author/s conducted the interview or focus group? | p13 |
| 2. Credentials | What were the researcher’s credentials? E.g. PhD, MD | p13 |
| 3. Occupation | What was their occupation at the time of the study? | p13 |
| 4. Gender | Was the researcher male or female? | p13 |
| 5. Experience and training | What experience or training did the researcher have? | p13 |
| *Relationship with participants* |  |  |
| 6. Relationship established | Was a relationship established prior to study commencement? | No |
| 7. Participant knowledge of the interviewer | What did the participants know about the researcher? e.g. personal goals, reasons for doing the research | Participants understood the reasons for doing the research (p7) |
| 8. Interviewer characteristics | What characteristics were reported about the inter viewer/facilitator? e.g. Bias, assumptions, reasons and interests in the research topic | p7 |
| **Domain 2: study design** |  |  |
| *Theoretical framework* |  |  |
| 9. Methodological orientation and Theory | What methodological orientation was stated to underpin the study? e.g. grounded theory, discourse analysis, ethnography, phenomenology, content analysis | n/a |
| *Participant selection* |  |  |
| 10. Sampling | How were participants selected? e.g. purposive, convenience, consecutive, snowball | p10-11 |
| 11. Method of approach | How were participants approached? e.g. face-to-face, telephone, mail, email | p11 |
| 12. Sample size | How many participants were in the study? | p15/Table 3 |
| 13. Non-participation | How many people refused to participate or dropped out? Reasons? | n/a |
| *Setting* |  |  |
| 14. Setting of data collection | Where was the data collected? e.g. home, clinic, workplace | p7-13 |
| 15. Presence of non-participants | Was anyone else present besides the participants and researchers? | n/a |
| 16. Description of sample | What are the important characteristics of the sample? e.g. demographic data, date | p15/Table 3 |
| *Data collection* |  |  |
| 17. Interview guide | Were questions, prompts, guides provided by the authors? Was it pilot tested? | p13-14 |
| 18. Repeat interviews | Were repeat inter views carried out? If yes, how many? | n/a |
| 19. Audio/visual recording | Did the research use audio or visual recording to collect the data? | n/a |
| 20. Field notes | Were ﬁeld notes made during and/or after the interview or focus group? | Yes |
| 21. Duration | What was the duration of the inter views or focus group? | p13 |
| 22. Data saturation | Was data saturation discussed? | n/a |
| 23. Transcripts returned | Were transcripts returned to participants for comment and/or correction? | No |
| **Domain 3: analysis and ﬁndings** |  |  |
| *Data analysis* |  |  |
| 24. Number of data coders | How many data coders coded the data? | Two |
| 25. Description of the coding tree | Did authors provide a description of the coding tree? | n/a |
| 26. Derivation of themes | Were themes identiﬁed in advance or derived from the data? | p14 |
| 27. Software | What software, if applicable, was used to manage the data? | p14 |
| 28. Participant checking | Did participants provide feedback on the ﬁndings? | No |
| *Reporting* |  |  |
| 29. Quotations presented | Were participant quotations presented to illustrate the themes/ﬁndings? Was each quotation identiﬁed? e.g. participant number | p16-18 |
| 30. Data and ﬁndings consistent | Was there consistency between the data presented and the ﬁndings? | Yes |
| 31. Clarity of major themes | Were major themes clearly presented in the ﬁndings? | p16-18 |
| 32. Clarity of minor themes | Is there a description of diverse cases or discussion of minor themes? | n/a |

**Supplementary Table 2 – STROBE Statement - checklist of items that should be included in reports of observational studies**

|  | Item No. | Recommendation | | | Page No. | |
| --- | --- | --- | --- | --- | --- | --- |
| **Title and abstract** | 1 | (*a*) Indicate the study’s design with a commonly used term in the title or the abstract | | | 1 | |
|  |  | (*b*) Provide in the abstract an informative and balanced summary of what was done and what was found | | | 2 | |
| Introduction | | | | | | |
| Background/rationale | 2 | Explain the scientific background and rationale for the investigation being reported | | | 4-5 | |
| Objectives | 3 | State specific objectives, including any prespecified hypotheses | | | 5 | |
| Methods | | | | | | |
| Study design | 4 | Present key elements of study design early in the paper | | | 6 | |
| Setting | 5 | Describe the setting, locations, and relevant dates, including periods of recruitment, exposure, follow-up, and data collection | | | 6-10 | |
| Participants | 6 | (*a*) *Cohort study*—Give the eligibility criteria, and the sources and methods of selection of participants. Describe methods of follow-up  *Case-control study*—Give the eligibility criteria, and the sources and methods of case ascertainment and control selection. Give the rationale for the choice of cases and controls  *Cross-sectional study*—Give the eligibility criteria, and the sources and methods of selection of participants | | | 8-9 | |
|  |  | (*b*) *Cohort study*—For matched studies, give matching criteria and number of exposed and unexposed  *Case-control study*—For matched studies, give matching criteria and the number of controls per case | | | n/a | |
| Variables | 7 | Clearly define all outcomes, exposures, predictors, potential confounders, and effect modifiers. Give diagnostic criteria, if applicable | | | 6-10 | |
| Data sources/ measurement | 8* | For each variable of interest, give sources of data and details of methods of assessment (measurement). Describe comparability of assessment methods if there is more than one group | | | 6-10 | |
| Bias | 9 | Describe any efforts to address potential sources of bias | | | 15 | |
| Study size | 10 | Explain how the study size was arrived at | | | 6-10 | |
| Quantitative variables | 11 | | Explain how quantitative variables were handled in the analyses. If applicable, describe which groupings were chosen and why | | 10 |  |
| Statistical methods | 12 | | (*a*) Describe all statistical methods, including those used to control for confounding | | 10 |  |
|  |  |  | (*b*) Describe any methods used to examine subgroups and interactions | | n/a |  |
|  |  |  | (*c*) Explain how missing data were addressed | | n/a |  |
|  |  |  | (*d*) *Cohort study*—If applicable, explain how loss to follow-up was addressed  *Case-control study*—If applicable, explain how matching of cases and controls was addressed  *Cross-sectional study*—If applicable, describe analytical methods taking account of sampling strategy | | n/a |  |
|  |  |  | (*e*) Describe any sensitivity analyses | | n/a |  |
| Results | | | | | |  |
| Participants | 13* | | (a) Report numbers of individuals at each stage of study—eg numbers potentially eligible, examined for eligibility, confirmed eligible, included in the study, completing follow-up, and analysed | | 11 |  |
|  |  |  | (b) Give reasons for non-participation at each stage | | n/a |  |
|  |  |  | (c) Consider use of a flow diagram | | n/a |  |
| Descriptive data | 14* | | (a) Give characteristics of study participants (eg demographic, clinical, social) and information on exposures and potential confounders | | 11, Table 3 |  |
|  |  |  | (b) Indicate number of participants with missing data for each variable of interest | | n/a |  |
|  |  |  | (c) *Cohort study*—Summarise follow-up time (eg, average and total amount) | | n/a |  |
| Outcome data | 15* | | *Cohort study*—Report numbers of outcome events or summary measures over time | | n/a |  |
|  |  |  | *Case-control study—*Report numbers in each exposure category, or summary measures of exposure | | n/a |  |
|  |  |  | *Cross-sectional study—*Report numbers of outcome events or summary measures | | 11 |  |
| Main results | 16 | | (*a*) Give unadjusted estimates and, if applicable, confounder-adjusted estimates and their precision (eg, 95% confidence interval). Make clear which confounders were adjusted for and why they were included | | 13 |  |
|  |  |  | (*b*) Report category boundaries when continuous variables were categorized | | n/a |  |
|  |  |  | (*c*) If relevant, consider translating estimates of relative risk into absolute risk for a meaningful time period | | n/a |  |
| Other analyses | 17 | | Report other analyses done—eg analyses of subgroups and interactions, and sensitivity analyses | n/a | |  |
| Discussion | | | | | |  |
| Key results | 18 | | Summarise key results with reference to study objectives | 13 | |  |
| Limitations | 19 | | Discuss limitations of the study, taking into account sources of potential bias or imprecision. Discuss both direction and magnitude of any potential bias | 15-16 | |  |
| Interpretation | 20 | | Give a cautious overall interpretation of results considering objectives, limitations, multiplicity of analyses, results from similar studies, and other relevant evidence | 13-16 | |  |
| Generalisability | 21 | | Discuss the generalisability (external validity) of the study results | 15-16 | |  |
| Other information |  | | | | |  |
| Funding | 22 | | Give the source of funding and the role of the funders for the present study and, if applicable, for the original study on which the present article is based | Acknowledgements | |  |

**Supplementary Table 3 - Modifications made to Rural Active Living Assessment (RALA) – Town-Wide Assessment**

| **Question no.** | **Original RALA Question** | **Final question** | **Modification made^*^** | **Rationale** |
| --- | --- | --- | --- | --- |
|  | Town demographics and characteristics | | | |
| X | Questions 1-8 – town demographics | Not applicable | Removed | Captured elsewhere by the research team to reduce participant burden |
| 1 | General town topography | Unchanged |  |  |
| 2 | Presence of “Town Center” | Presence of “Town Centre” | “center” changed to “centre”  In response options, definitions provided for “Distinct town centre” defined as “where all or most of the town municipal & commercial services are located, such as town offices, library, police/fire departments, post office” and “Multiple town centres” defined as “more than one area is considered the town centre; municipal & commercial services found in both/all of these “centres”) | Local context  For clarity |
| 3 | General town street pattern | Unchanged | In response options definitions provided for “Grid” defined as “roads have an obvious and distinguishable grid pattern in one or several sections of the town; grid patterns are similar to those found in urban areas with distinct city blocks” and “Radial” defined as “where primary roads generally intersect and come together at a central point; this may look like the spokes of a tyre” | For clarity |
| 4 | Location of public high school | Location of nearest public high school | “nearest” included | For clarity |
| X | Location of public middle school | Not applicable | Removed | Not suited to local context |
| 5 | Local of public elementary school | Location of nearest public primary school | “nearest” included  “elementary” changed to “primary” | For clarity  Local context |
| 6 | Not applicable | Location of nearest early learning or childcare (long daycare) centre | Added new question | Local context |
| 7 | Location of any additional school | Are there additional schools/learning institutions nearby? | A few wording changes including adding “learning institutions” and “nearby”  In response options, added “early learning/childcare centre”, “(years 7-10)” to “public high school”, “public college (years 11-12)” and “TAFE” to “university” instead of “college”. Changed “elementary” to “primary” | For clarity and completeness  Local context |
|  | Town recreational amenities | | | |
| 8 | Hiking or walking trail(s)^ | Unchanged | Not applicable | Not applicable |
| 9 | Not applicable | How many hiking or walking trails do you have in your town? | New question | For completeness |
| 10 | Biking path(s)^ | Biking path(s)/trails(s) | Added “trail(s)” | To capture unpaved and multi-user trails (e.g. rail trails) |
| 11 | Not applicable | How many bike paths do you have in your town? | New question | For completeness |
| 12 | Public park(s)^ | Unchanged | Added to response options “within 500 m of town centre” | Local context |
| 13 | Not applicable | Are there public toilets available in your town?^ | New question | Local context and identified need in pilot study |
| 14 | Swimming beach^ | Unchanged | Not applicable | Not applicable |
| 15 | Public use swimming pool^ | Unchanged | Not applicable | Not applicable |
| 16 | Not applicable | Lake for swimming or with canoe/kayak/boat/watersport access^ | New question | For completeness |
| 17 | River with canoe/kayak/boat/watersport access^ | River for swimming or with canoe/kayak/boat/watersport access | Added “swimming” | Local context |
| 18 | Skate park^ | Skate/BMX park | Added “BMX” | Local context |
| X | Ice skating rink | Not applicable | Removed | Not suited to local context |
| X | Roller skating rink | Not applicable | Removed | Not suited to local context |
| 19 | Town/recreation center (e.g. YMCA or town recreation facility)^ | Town/recreation centre (e.g. YMCA or town recreation facility/hall) | Added “hall” “center” changed to “centre” | Local context |
| 20 | Private fitness facility (e.g. Curves, Gold’s Gym etc)^ | Private gym/fitness centre (e.g. Zap Fitness, Fernwood etc) | “Fitness facility” changed to “gym/fitness centre” and different examples provided | Local context |
| 21 | Playgrounds^ | Unchanged | Added to response options “within 500 m of town centre” | Local context |
| 22 | Playing fields or courts^ | Playing fields (e.g. soccer, AFL, baseball, hockey) | Separated fields from courts and provided examples | Local context |
| 23 | Not applicable | Indoor or outdoor courts (e.g. netball, basketball, tennis, squash, badminton)^ | New question to separate fields and courts | Local context |
| 24 | Other amenity | Are there other amenities?^ | Some wording changes | For clarity |
|  |  | | | |
| 25 | Not applicable | Do you have any other comments to make on the town’s amenities or facilities? | New question | For completeness |

^*^where relevant, mile was changed to metre or kilometre in response options

^ Where these amenities existed, a further question was asked about the condition of the amenity, rated as Fair/Poor or Good/Excellent

**Supplementary Table 4** - Modifications made to Rural Active Living Assessment (RALA) – Program and Policy Assessment

| **Question no.** | **Original RALA Question** | **Final question** | **Modification made** | **Rationale** |
| --- | --- | --- | --- | --- |
|  | Town Programs and Policies | | | |
| 1 | Does the town have a policy that requires bikeways or pedestrian walkways in new public infrastructure projects? | Does the town/council have a policy that requires bikeways or pedestrian walkways in new public infrastructure projects? | Added “council” | Local context – typically these policies in Australia are made at the council rather than town level |
| 2 | Does the town regularly clear snow from sidewalks? | Does the town/council regularly clear obstacles from footpaths? (e.g. branches, leaves, dirt after drain overflow) | Several wording changes | Local context – due to Australia’s warmer climate, snow clearing is not a common activity; ‘sidewalks’ are more commonly referred to as ‘footpaths’ |
| 3 | Does the town have a public recreation department that offers physical activity programming? | Does the town/council have a community recreation department that offers physical activity programming? | Added “council”. Changed “public” to “community”.   Removed subsequent questions available when primary question responded to as “Yes” | Local context.    Sub-questions poorly answered/deemed unhelpful in pilot study |
| 4 | Does the town have a private organization (such as the YMCA or a religious organization) that offers physical activity programming? | Are physical activity resources/facilities available for local resident use outside of programs? | Multiple changes made | Local context  Primary question and sub-questions poorly answered/deemed unhelpful in pilot study |
| X | Does the town offer any *local* public transportation options, such as public busses or vans? | Not applicable | Removed | Question poorly answered/not helpful in pilot study, often due to small size of towns with no public transport options |
| X | Are there any *long-distance* public transportation options available in your town, such as a train or Greyhound Bus? | Not applicable | Removed | Question poorly answered/deemed unhelpful in pilot study |
|  | School Programs and Policies | | | |
| 5 | Does the town have any “Walk to School” programs or other programs that encourage children to walk or bike to school? | Does the town have any “Walk to School” programs or other programs that encourage children to walk or bike to school? | Unchanged |  |
| X | Does the town participate in the National “Safe Routes to School” program? | Not applicable | Removed | Not suited to local context |
| X | Do the public schools in the town offer other sponsored physical activity initiatives for students? | Not applicable | Removed | Question poorly answered/deemed unhelpful in pilot study |
| 6 | Do the public schools in the town allow public access to their recreation facilities after school hours? | Do the schools in the town allow public access to their recreation facilities after school hours? | “public schools” changed to “schools” | Local context |
| X | Do the public schools have a late bus option for children that stay after school for sponsored activities? | Not applicable | Removed | Not suited to local context  Question poorly answered/deemed unhelpful in pilot study |
| X | What percent (%) of children live within 1-mile of their school? | Not applicable | Removed | Not suited to local context |
|  |  | | | |
| 7 | Not applicable | Do you have any other comments to make about the policies or programs in the town that support walking and being active? | Added new question | To ensure all relevant information captured |

**Supplementary Table 5** - Modifications made to Rural Active Living Assessment (RALA) – Segment Assessment

| **Question no.** | **Original RALA Question** | **Final question** | **Modification made^*^** | **Rationale** |
| --- | --- | --- | --- | --- |
| X | Questions 1-4 | Not applicable | Removed | Captured elsewhere by research team to reduce participant burden |
|  | Primary land use & terrain | | | |
| 1 | Land use | Unchanged | Added to response options “mixed use” | Local context |
| 2 | Terrain | Unchanged | Not applicable | Not applicable |
| 3 | Segment zone type | Unchanged | Not applicable | Not applicable |
|  | Walkability | | | |
| 4 | Sidewalks | Footpaths (paved)  Footpaths (unpaved) | Sidewalks changed to “footpaths”  Separate question for “paved” and “unpaved” footpaths  “Footpath only” removed from response options | Local context     For clarity |
| 5 | Not applicable | Trees for shade | New question | Presence of shade influences walkability |
| 6 | Buffers and shoulders | Unchanged | “Sidewalk” changed to “footpath” in response options | Local context |
| 7 | Cross walks and pedestrian signage | Zebra crossings and pedestrian signage | “Cross walks” changed to “Zebra crossing” | Local context |
| 8 | Other safety features | Unchanged | Added to response options “slow down signs”, “school crossing guides”, “chicanes”, “ramps on footpath gutters” | Local context |
| 9 | Road/traffic characteristics | Unchanged | Not applicable | Not applicable |
| 10 | Barriers | Unchanged | In response options, changed: “highway” to “highway crosses over/impacts; “train tracks” to “train tracks cross over/impacts”; “private property – no trespassing” to “private property (no trespassing) impacts”. Added “roundabout” | For clarity and local context |
| 11 | Connectivity:  Do sidewalks, a bikepath or other trail link this segment to other parts of town / attractions or to another segment or road? | Connectivity:  Do footpaths, bikepaths or other trails link this segment to other parts of town / attractions or to  another segment or road? | “Sidewalks” changed to “footpaths” | Local context |
|  | Land use | | | |
| 12 | Residential | Residential land use | Minor wording change  In response options, changed “mobile homes” to “motorhomes or caravans”  Removed “condition” rating | For clarity  Local context    Limited salience |
| 13 | Public/civic | Public/civic land use | Minor word change  In response options, added: “LINC” to library”, “online access centre”, “community hall”, “men’s shed”, “public toilet”, “other public open space”. Changed “courthouse” to “court of law”, “athletic fields/courts” to “sports fields” and “indoor/outdoor sports courts”, “playground” to “public park/pocket park/playground”  Removed “condition” rating | For clarity  Local context  Limited salience |
| 14 | Commercial | Commercial land use | Minor wording change  In response options, added: “small food store (e.g. bakery, butcher, fruit shop)”, “farmers market”. Changed “bar” to “pub/bar/nightclub”, “food market” to “supermarket”, “theater” to “theatre/cinema”, “gas station” to “petrol station”, “convenience store” to “convenience/general store”, “small retail” to “small retail shop”, “big box retail” to “large retail shop (e.g. Kmart)”, “fitness center” to “gym/fitness centre”  Removed “condition” rating | For clarity  Local context  Limited salience |
| 15 | Schools | Unchanged | In response options, changed “elementary” to “primary”. Added “early learning/childcare centre”. Removed “middle (public)”  Removed “condition” rating | Local context      Limited salience |
| 16 | Industrial agriculture | Unchanged | Removed “condition” rating | Limited salience |
|  | Subjective assessment | | | |
| 17 | How strongly do you agree with the following statement? “*This segment is walkable”* | How much do you agree or disagree with the following statement? “This segment is walkable.” | Minor wording change | For clarity |
| 18 | How strongly do you agree with the following statement? “*This segment is aesthetically pleasing”* | How much do you agree or disagree with the following statement? “This  segment is attractive to look at and pleasant to be in.” | Several wording changes. Question positioned at end of this section | For clarity |
| 19 | Not applicable | How much do you agree or disagree with the following statement? “This  segment is walkable for an older person, someone with limited mobility or a pram.” | New question | For greater inclusion based on feedback from pilot study |
| 20 | Not applicable | How much do you agree or disagree with the following statement? “This segment is walkable for a child around the age of 10 to walk on their own.” | New question | For greater inclusion based on feedback from pilot study |
|  | General conditions | | | |
| 21 | Current weather conditions | Unchanged | Moved from end of tool to after “Primary land use and terrain” section | To improve flow |
| 22 | Season | Unchanged | Moved from end of tool to after “Primary land use and terrain” section | To improve flow |
| 23 | Day of week | Unchanged | Moved from end of tool to after “Primary land use and terrain” section | To improve flow |
|  |  | | | |
| 24 | Not applicable | Do you have any other comments to make about this segment? | New question | For completeness |

**Supplementary Table 6: Descriptive characteristics of segments and audit conditions from the C4W digital tool (n=80)**

The most common land use for the town segments were mixed-use, around half of the segments were neighbourhood zones, the terrain was most often mixed or flat, and few had schools. Most assessments were conducted on sunny/clear or partly cloudy days, across all seasons but most commonly in autumn, and on weekdays.

| **Characteristic** | **Details** |
| --- | --- |
| **Land use, n (%)** |  |
| Residential | 22 (27.5%) |
| Commercial | 1 (1.3%) |
| Industrial | 0 |
| Open space | 2 (2.5%) |
| Public/civic | 5 (6.3%) |
| Mixed use | 49 (61.3%) |
| Other | 0 |
| Not reported | 1 (1.3%) |
| **Segment zone, n (%)** |  |
| Town centre | 11 (13.8%) |
| Thoroughfare | 6 (7.5%) |
| Neighbourhood | 40 (50.0%) |
| Isolated school zone | 0 |
| Mixed | 20 (25%) |
| Not reported | 3 (3.8%) |
| **Terrain, n (%)** |  |
| Flat | 33 (41.3%) |
| Wooded/undeveloped | 0 |
| Hills | 7 (8.8%) |
| Water body | 0 |
| Winding roads | 3 (3.8%) |
| Mixed | 32 (40%) |
| Other | 2 (2.5%) |
| Not reported | 3 (3.8%) |
| **Schools** |  |
| Early learning/childcare centre | 0 |
| Public primary | 10 (12.5%) |
| Public secondary | 0 |
| Private school | 0 |
| Other | 1 (1.3%) |
| Mixed | 6 (7.5%) |
| No school | 63 (78.8%) |
| **Weather conditions** |  |
| Sunny/clear | 28 (35.0%) |
| Partly cloudy | 27 (33.8%) |
| Overcast | 12 (15.0%) |
| Rain | 1 (1.3%) |
| Other | 5 (6.3%) |
| Not reported | 7 (8.8%) |
| **Season** |  |
| Summer | 12 (15.0%) |
| Autumn | 35 (43.8%) |
| Winter | 10 (12.5%) |
| Spring | 16 (20.0%) |
| Not reported | 7 (8.8%) |
| **Day of week** |  |
| Weekday | 46 (57.5%) |
| Weekend | 24 (30.0%) |
| Holiday | 3 (3.8%) |
| Not reported | 7 (8.8%) |

**Supplementary Table 7: Summary measures of land use derived from the C4W digital tool (n=80 segments)**

Residential land use mainly consisted of single-family detached homes (63%) and were moderately densely settled. Around half of the segments were industrial/agricultural, with most identified as farmland. Just under half of all segments had commercial land uses, most commonly mixed, and public/civic uses also tended to be mixed.

| **Residential land use** |  |
| --- | --- |
| Single family detached homes | 50 (62.5%) |
| Multi-family homes / apartments | 3 (3.8%) |
| Mixed | 13 (16.3%) |
| Other | 3 (3.8%) |
| None | 11 (13.8%) |
| **Residential density** |  |
| Densely settled | 3 (3.8%) |
| Moderately densely settled | 43 (53.8%) |
| Not densely settled (dispersed) | 23 (28.8%) |
| None | 11 (13.8%) |
| **Industrial/agricultural land use** |  |
| Farmland area | 17 (21.3%) |
| Light industrial area | 4 (5.0%) |
| Heavy industrial area | 2 (2.5%) |
| Mixed | 7 (8.8%) |
| Other | 7 (8.8%) |
| None | 43 (53.8%) |
| **Commercial land use** |  |
| Convenience/General store | 1 (1.3%) |
| Farmer’s market | 1 (1.3%) |
| Pub/bar/nightclub | 1 (1.3%) |
| Restaurant / café | 3 (3.8%) |
| Small retail shop | 1 (1.3%) |
| Mixed | 27 (33.8%) |
| Other | 3 (3.8%) |
| None | 43 (53.8%) |
| **Public/civic land use** |  |
| Church/religious | 1 (1.3%) |
| Other public open space | 4 (5.0%) |
| Public toilet | 1 (1.3%) |
| Sports field | 1 (1.3%) |
| Other | 10 (12.5%) |
| Mixed | 41 (51.3%) |
| None | 22 (27.5%) |

**Supplementary Table 8: Individual items from the online segment audits used to create summary measures**

| **Footpath buffer** |  |
| --- | --- |
| Good/excellent | 16 (20.0%) |
| Poor/fair | 36 (45.0%) |
| None | 28 (35.0%) |
| **Defined curb shoulder** |  |
| Good/excellent | 17 (21.3%) |
| Poor/fair | 40 (50.0%) |
| None | 23 (28.8%) |
| **Zebra crossing** |  |
| Good/excellent | 2 (2.5%) |
| Poor/fair | 9 (11.3%) |
| None | 69 (86.3%) |
| **Crossing signals** |  |
| Good/excellent | 1 (1.3%) |
| Poor/fair | 13 (16.3%) |
| None | 66 (82.6%) |
| **Pedestrian signs** |  |
| Good/excellent | 6 (7.5%) |
| Poor/fair | 13 (16.3%) |
| None | 61 (76.3%) |
| **Children at play signs** |  |
| Good/excellent | 1 (1.3%) |
| Poor/fair | 12 (15.0%) |
| None | 67 (83.8%) |
| **Other safety features** |  |
| Public lighting | 5 (6.3%) |
| Ramps on footpath gutters | 7 (8.8%) |
| School crossing guides | 1 (1.3%) |
| School flashing lights | 1 (1.3%) |
| Slow down signs | 4 (5.8%) |
| Speed bumps | 1 (1.3%) |
| Stop signs | 1 (1.3%) |
| Mixed | 24 (30%) |
| None | 36 (45.0%) |
| **Barriers** |  |
| Highway crosses over/impacts | 5 (6.3%) |
| Industrial zone | 1 (1.3%) |
| Natural features | 10 (12.5%) |
| Private property (no trespassing) impacts | 1 (1.3%) |
| Roundabout | 1 (1.3%) |
| Train tracks cross over/impacts | 1 (1.3%) |
| Other | 4 (5.0%) |
| Mixed | 13 (16.3%) |
| None | 44 (55.0%) |
| **Tree shade** |  |
| Both sides | 3 (3.8%) |
| One side | 6 (7.5%) |
| Intermittent | 43 (53.8%) |
| None | 20 (25.0%) |
| Not reported | 8 (10.0%) |
| **Road type** |  |
| Paved multi-lane roads | 28 (35.0%) |
| Paved single lane roads | 40 (50.0%) |
| Unpaved roads | 4 (5.0%) |
| Not reported | 8 (10.0%) |
| **Road condition** |  |
| Good/excellent | 47 (58.8%) |
| Poor/fair | 25 (31.3%) |
| Not reported | 8 (10.0%) |
| **Traffic volume** |  |
| High | 10 (12.5%) |
| Medium | 25 (31.3%) |
| Low | 37 (46.3%) |
| Not reported | 8 (10.0%) |
| **Connectivity** |  |
| Yes | 47 (58.8%) |
| No | 24 (30.0%) |
| Not reported | 9 (11.3%) |
| **Footpaths – paved - Location** |  |
| Both sides of street | 7 (8.8%) |
| One side of street | 25 (31.3%) |
| Intermittent | 12 (15.0%) |
| No footpath | 28 (35.0%) |
| Not reported | 8 (10.0%) |
| **Footpaths – paved - Condition** |  |
| Good/excellent | 30 (37.5%) |
| Poor/fair | 15 (18.8%) |
| Not reported | 35 (43.8%) |
| **Footpaths – unpaved - Location** |  |
| Both sides of street | 4 (5.0%) |
| One side of street | 23 (28.8%) |
| Intermittent | 17 (21.3%) |
| No footpath | 27 (33.8%) |
| Not reported | 9 (11.3%) |
| **Footpaths – unpaved - Condition** |  |
| Good/excellent | 13 (16.3%) |
| Poor/fair | 31 (38.8%) |
| Not reported | 36 (45.0%) |
| **Overall walkability** |  |
| Strongly agree | 12 (15.0%) |
| Agree | 38 (47.5%) |
| Disagree | 15 (18.8%) |
| Strongly disagree | 6 (7.5%) |
| Not reported | 9 (11.3%) |
| **Walkability accessibility** |  |
| Strongly agree | 6 (7.5%) |
| Agree | 23 (28.8%) |
| Disagree | 23 (28.8%) |
| Strongly disagree | 19 (23.8%) |
| Not reported | 9 (11.3%) |
| **Walkability for children** |  |
| Strongly agree | 7 (8.8%) |
| Agree | 21 (26.3%) |
| Disagree | 22 (27.5%) |
| Strongly disagree | 21 (26.3%) |
| Not reported | 9 (11.3%) |
| **Aesthetics** |  |
| Strongly agree | 20 (25.0%) |
| Agree | 36 (45.0%) |
| Disagree | 12 (15.0%) |
| Strongly disagree | 3 (3.8%) |
| Not reported | 9 (11.3%) |

*Comparison of data from the C4W digital tool and geospatially assessed walkability index*

Supplementary Table 9 lists Spearman correlation estimates, 95% confidence intervals (CIs) and p-values, along with the corresponding Supplementary Figure.

**Supplementary Table 9: Estimates, confidence intervals and p-values for geospatially assessed walkability decile and various audit variables**

| **Dependent variable** | **Independent variable** | **Correlation estimate (r)** | **95% confidence interval (CI)** | **p-value** | **Supplementary Figure** |
| --- | --- | --- | --- | --- | --- |
| Decile | Overall walkability | 0.44 | -0.22-0.82 | 0.18 | 1 |
| Decile | Aesthetics | 0.07 | -0.55-0.65 | 0.83 | 1 |
| Decile | Accessibility for older adults | 0.43 | -0.23-0.82 | 0.18 | 1 |
| Decile | Accessibility for children | 0.21 | -0.45-0.71 | 0.54 | 1 |
| Decile | All walkability scores (mean) | 0.22 | -0.44-0.73 | 0.51 | 1 |
| Decile | Public land use features | 0.33 | -0.34-0.78 | 0.32 | 2 |
| Decile | Commercial land use features | 0.61 | 0.01-0.88 | <0.05* | 2 |
| Decile | All land use features combined | 0.37 | -0.29-0.80 | 0.26 | 2 |
| Decile | Buffers and shoulders | 0.40 | -0.27-0.80 | 0.23 | 3 |
| Decile | Crossing and signage | 0.23 | -0.43-0.73 | 0.23 | 3 |
| Decile | ‘Other’ safety features | 0.71 | 0.18-0.92 | <0.05* | 3 |
| Decile | All safety features | 0.48 | -0.17-0.84 | 0.14 | 3 |
| Decile | Tree shade | 0.25 | -0.41-0.74 | 0.46 | 4 |
| Decile | Footpaths | 0.41 | -0.26-0.81 | 0.22 | 4 |
| Decile | Traffic volume | 0.00 | -0.60-0.60 | 1 | 4 |
| Decile | Connectivity | 0.38 | -0.29-0.80 | 0.25 | 4 |
| Decile | Enablers | 0.46 | -0.19-0.83 | 0.15 | 4 |
| Decile | Barriers | 0.18 | -0.47-0.70 | 0.60 | 4 |
| Decile | Total barriers and enablers | 0.39 | -0.27-0.80 | 0.24 | 4 |
| Decile | Town wide assessment | 0.33 | -0.42-0.82 | 0.39 | 5 |
| Town population | Town wide assessment | -0.73 | -0.94--0.13 | <0.05* | 5 |
| Decile | Policy and program assessment | 0.15 | -0.57-0.74 | 0.7 | 6 |

* <0.05

**Supplementary Figure 1: Correlation (Spearman) between audit accessibility and walkability scores and geospatially-assessed walkability scores (n=11)**


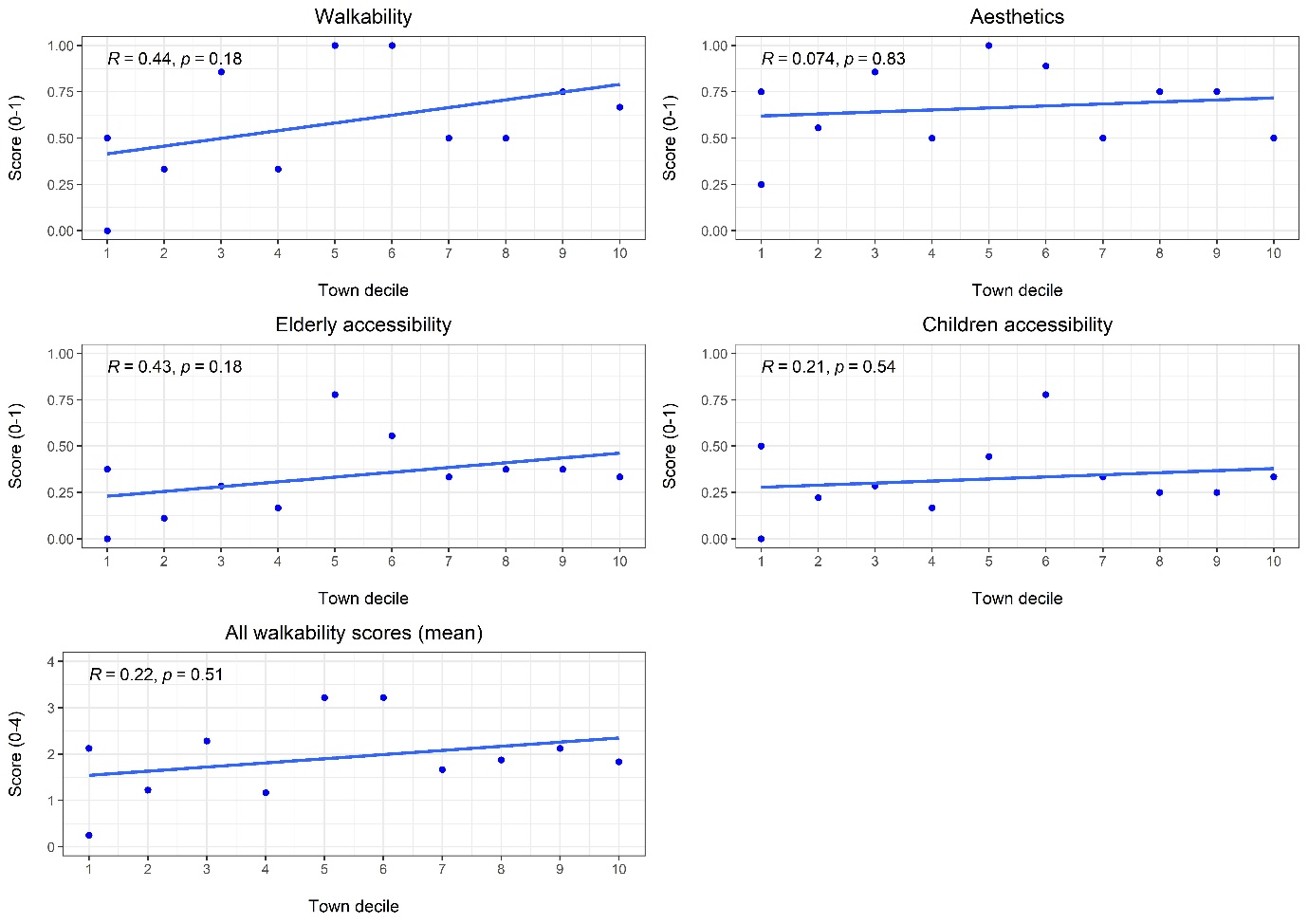


**Supplementary Figure 2: Correlation (Spearman) between audit land use scores and geospatially-assessed walkability scores (n=11)**


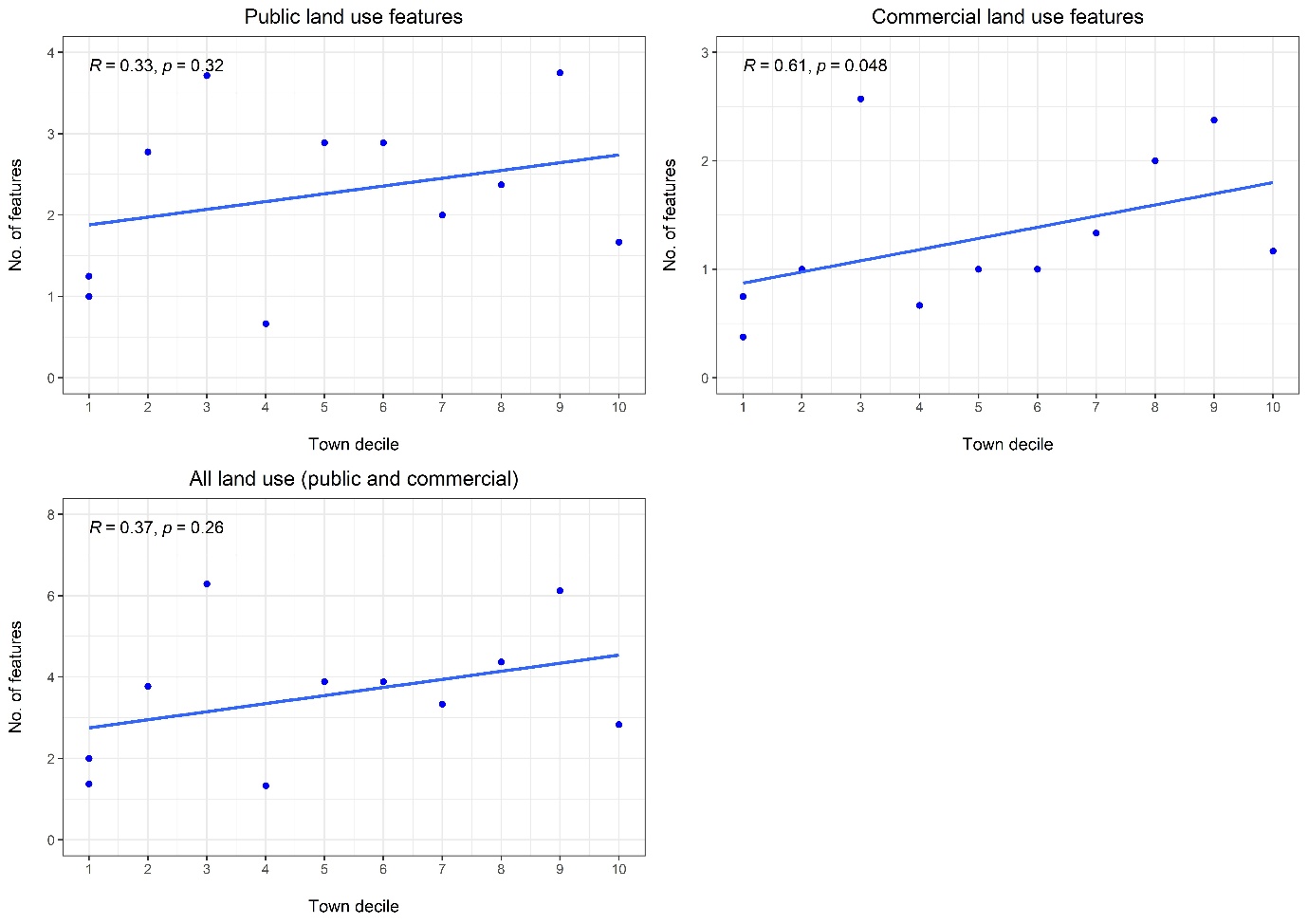


**Supplementary Figure 3: Correlation (Spearman) between audit safety scores and geospatially-assessed walkability scores (n=11)**


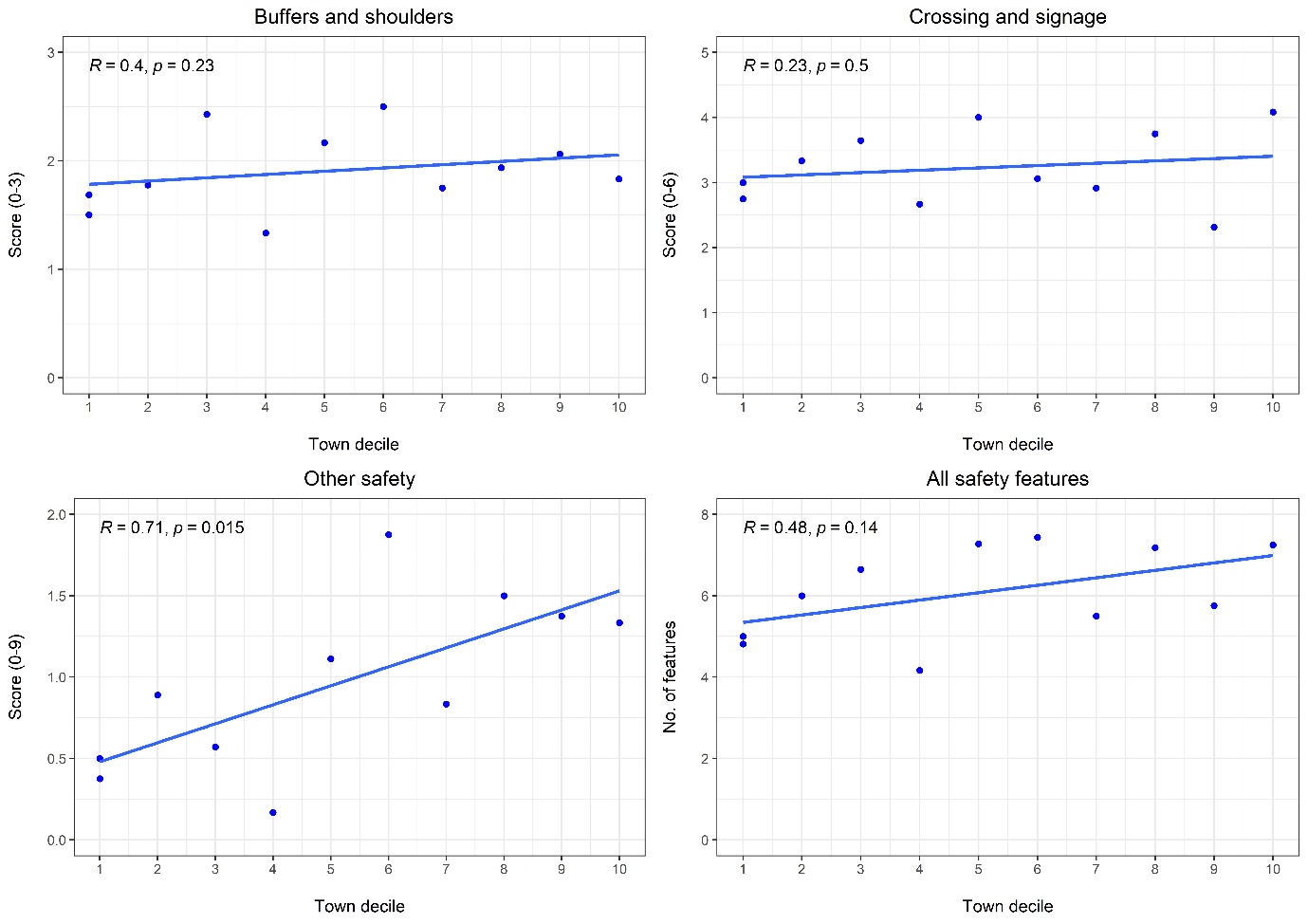


**Supplementary Figure 4: Correlation (Spearman) between audit barriers and enablers scores and geospatially-assessed walkability scores (n=11)**


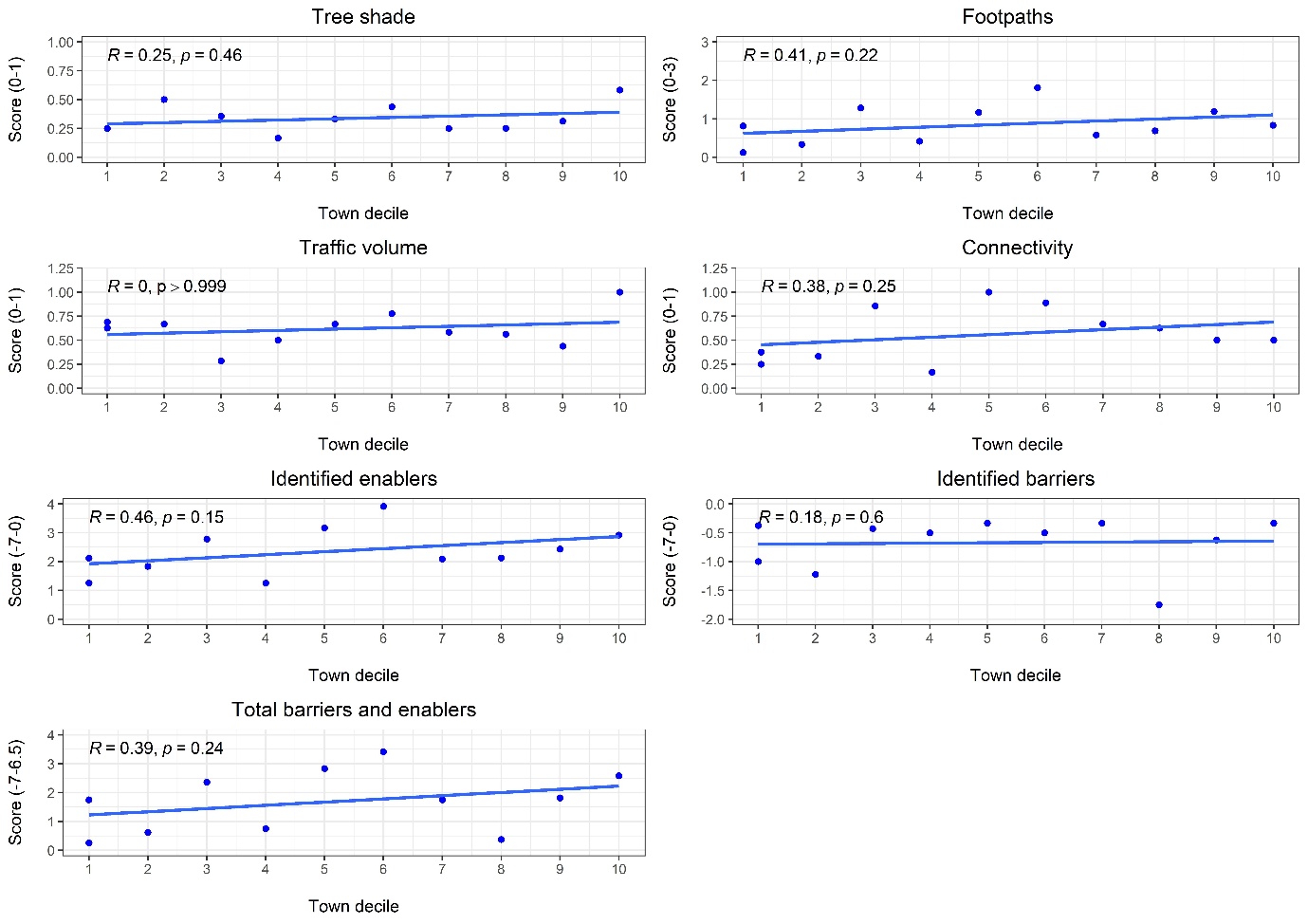


**Supplementary Figure 5 – Correlation (Spearman) between townwide assessments and geospatially-assessed walkability scores (n=9)**


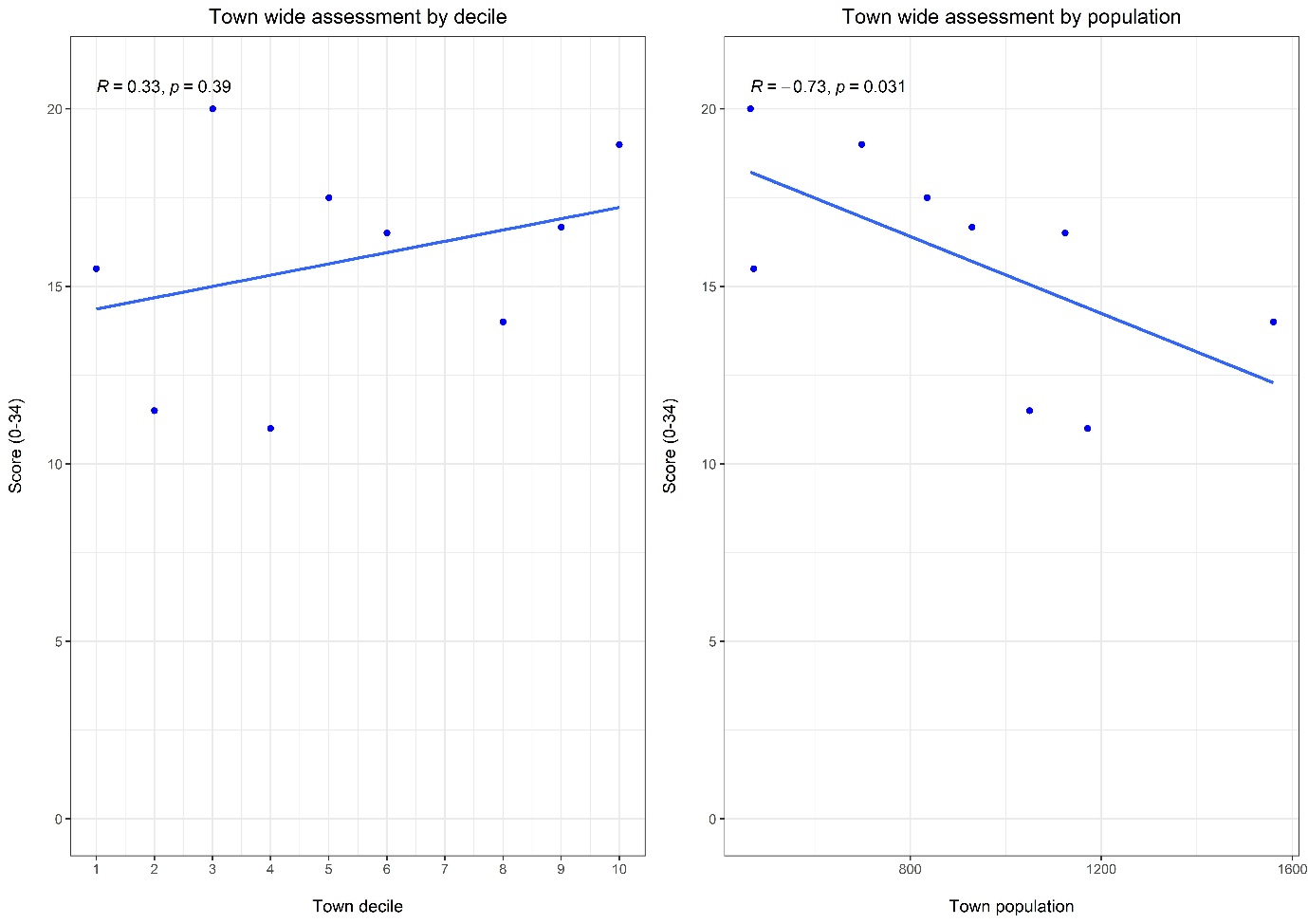


**Supplementary Figure 6 – Correlation (Spearman) between policy and program characteristics and geospatially-assessed walkability scores (n=9)**


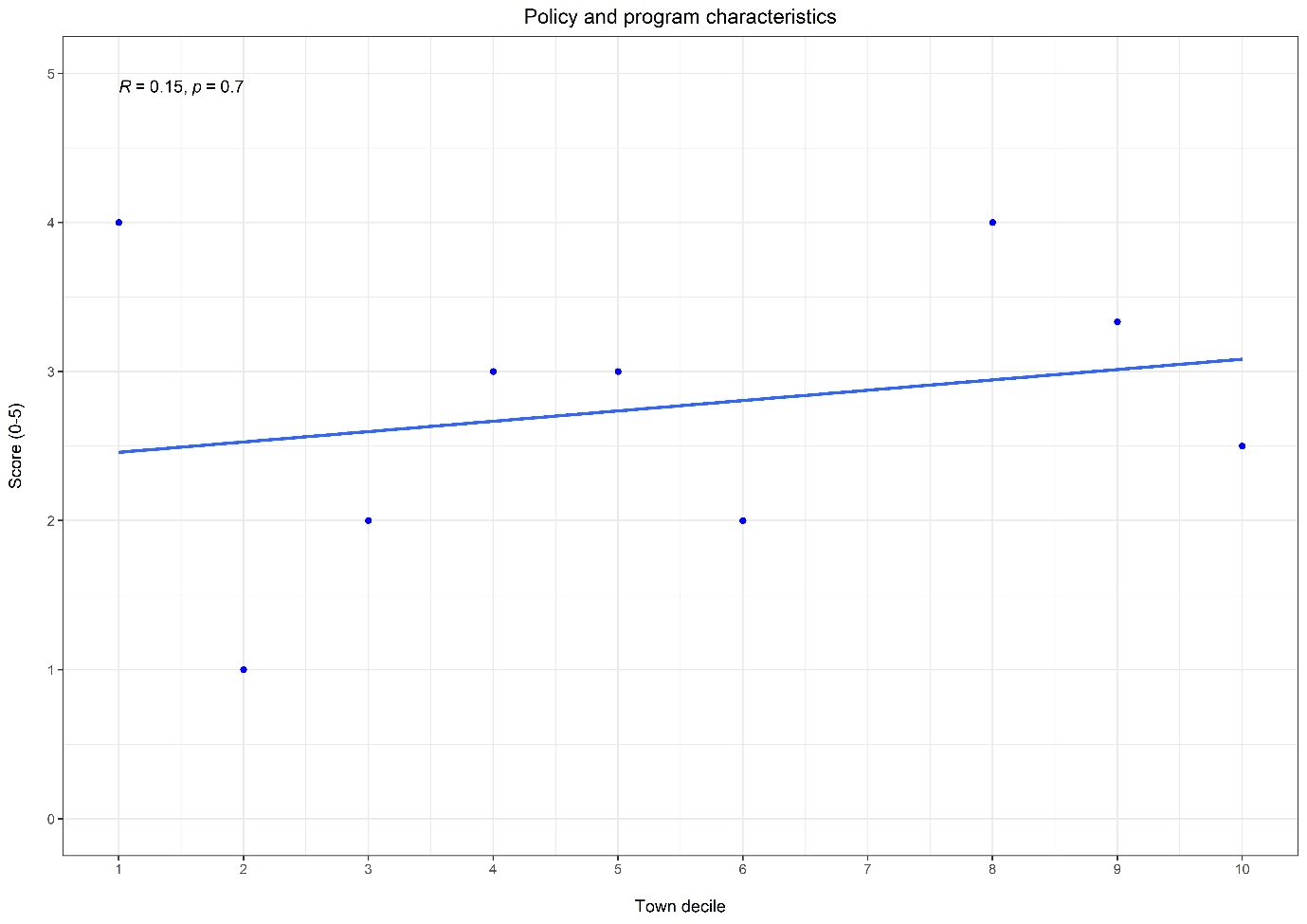

Supplement: Supplementary file 1 — Supporting File [file HEX-29-e70661-s001.docx]
